# Supplementary material for: Resolving the full spectrum of human genome variation using Linked-Reads
Source: Genome Res. 2019 Apr;29(4):635–45. doi: 10.1101/gr.234443.118 (PMC6442396; doi:10.1101/gr.234443.118)
Supplement: Supplemental Material [file supp_gr.234443.118_Supplemental_longranger-2.2.zip › longranger-2.2.tar.bz2!/lr2.2-public/lib/stan/cmdstan/stan/doc/api/html/namespacestan_1_1model.html]

Stan: stan::model Namespace Reference


|  |  |
| --- | --- |
|  | Stan  2.5.0  probability, sampling & optimization |


- Main Page
- Related Pages
- Namespaces
- Classes
- Files

- Namespace List
- Namespace Members

All Classes Namespaces Files Functions Variables Typedefs Enumerations Enumerator Friends Macros Pages

- stan
- model

Classes |
Functions

stan::model Namespace Reference

For compiling models.
More...

|  |  |
| --- | --- |
| Classes | |
| class | prob\_grad |
|  | The `prob_grad` class represents the basic parameter holders for a model. More... |
|  | |
| struct | model\_functional |
|  | |

|  |  |
| --- | --- |
| Functions | |
| template<bool jacobian\_adjust\_transform, class M > | |
| double | log\_prob\_propto (const M &model, std::vector< double > &params\_r, std::vector< int > &params\_i, std::ostream \*msgs=0) |
|  | Helper function to calculate log probability for `double` scalars up to a proportion. More... |
|  | |
| template<bool propto, bool jacobian\_adjust\_transform, class M > | |
| double | log\_prob\_grad (const M &model, std::vector< double > &params\_r, std::vector< int > &params\_i, std::vector< double > &gradient, std::ostream \*msgs=0) |
|  | Compute the gradient using reverse-mode automatic differentiation, writing the result into the specified gradient, using the specified perturbation. More... |
|  | |
| template<bool jacobian\_adjust\_transform, class M > | |
| double | log\_prob\_propto (const M &model, Eigen::VectorXd &params\_r, std::ostream \*msgs=0) |
|  | Helper function to calculate log probability for `double` scalars up to a proportion. More... |
|  | |
| template<bool propto, bool jacobian\_adjust\_transform, class M > | |
| double | log\_prob\_grad (const M &model, Eigen::VectorXd &params\_r, Eigen::VectorXd &gradient, std::ostream \*msgs=0) |
|  | Compute the gradient using reverse-mode automatic differentiation, writing the result into the specified gradient, using the specified perturbation. More... |
|  | |
| template<bool propto, bool jacobian\_adjust\_transform, class M > | |
| void | finite\_diff\_grad (const M &model, std::vector< double > &params\_r, std::vector< int > &params\_i, std::vector< double > &grad, double epsilon=1e-6, std::ostream \*msgs=0) |
|  | Compute the gradient using finite differences for the specified parameters, writing the result into the specified gradient, using the specified perturbation. More... |
|  | |
| template<bool propto, bool jacobian\_adjust\_transform, class M > | |
| int | test\_gradients (const M &model, std::vector< double > &params\_r, std::vector< int > &params\_i, double epsilon=1e-6, double error=1e-6, std::ostream &o=std::cout, std::ostream \*msgs=0) |
|  | Test the log\_prob\_grad() function's ability to produce accurate gradients using finite differences. More... |
|  | |
| template<bool propto, bool jacobian\_adjust\_transform, class M > | |
| double | grad\_hess\_log\_prob (const M &model, std::vector< double > &params\_r, std::vector< int > &params\_i, std::vector< double > &gradient, std::vector< double > &hessian, std::ostream \*msgs=0) |
|  | Evaluate the log-probability, its gradient, and its Hessian at params\_r. More... |
|  | |
| template<class M > | |
| void | gradient (const M &model, const Eigen::Matrix< double, Eigen::Dynamic, 1 > &x, double &f, Eigen::Matrix< double, Eigen::Dynamic, 1 > &grad\_f, std::ostream \*msgs=0) |
|  | |
| template<class M > | |
| void | hessian (const M &model, const Eigen::Matrix< double, Eigen::Dynamic, 1 > &x, double &f, Eigen::Matrix< double, Eigen::Dynamic, 1 > &grad\_f, Eigen::Matrix< double, Eigen::Dynamic, Eigen::Dynamic > &hess\_f, std::ostream \*msgs=0) |
|  | |
| template<class M > | |
| void | gradient\_dot\_vector (const M &model, const Eigen::Matrix< double, Eigen::Dynamic, 1 > &x, const Eigen::Matrix< double, Eigen::Dynamic, 1 > &v, double &f, double &grad\_f\_dot\_v, std::ostream \*msgs=0) |
|  | |
| template<class M > | |
| void | hessian\_times\_vector (const M &model, const Eigen::Matrix< double, Eigen::Dynamic, 1 > &x, const Eigen::Matrix< double, Eigen::Dynamic, 1 > &v, double &f, Eigen::Matrix< double, Eigen::Dynamic, 1 > &hess\_f\_dot\_v, std::ostream \*msgs=0) |
|  | |
| template<class M > | |
| void | grad\_tr\_mat\_times\_hessian (const M &model, const Eigen::Matrix< double, Eigen::Dynamic, 1 > &x, const Eigen::Matrix< double, Eigen::Dynamic, Eigen::Dynamic > &X, Eigen::Matrix< double, Eigen::Dynamic, 1 > &grad\_tr\_X\_hess\_f, std::ostream \*msgs=0) |
|  | |

## Detailed Description

For compiling models.

## Function Documentation

template<bool propto, bool jacobian\_adjust\_transform, class M >

|  |  |  |  |
| --- | --- | --- | --- |
| void stan::model::finite\_diff\_grad | ( | const M & | *model*, |
|  |  | std::vector< double > & | *params\_r*, |
|  |  | std::vector< int > & | *params\_i*, |
|  |  | std::vector< double > & | *grad*, |
|  |  | double | *epsilon* = `1e-6`, |
|  |  | std::ostream \* | *msgs* = `0` |
|  | ) |  |  |

Compute the gradient using finite differences for the specified parameters, writing the result into the specified gradient, using the specified perturbation.

Template Parameters
:   |  |  |
    | --- | --- |
    | propto | True if calculation is up to proportion (double-only terms dropped). |
    | jacobian\_adjust\_transform | True if the log absolute Jacobian determinant of inverse parameter transforms is added to the log probability. |
    | M | Class of model. |

Parameters
:   |  |  |  |
    | --- | --- | --- |
    |  | model | Model. |
    |  | params\_r | Real-valued parameters. |
    |  | params\_i | Integer-valued parameters. |
    | [out] | grad | Vector into which gradient is written. |
    |  | epsilon |  |
    | [in,out] | msgs |  |

Definition at line 217 of file util.hpp.

template<bool propto, bool jacobian\_adjust\_transform, class M >

|  |  |  |  |
| --- | --- | --- | --- |
| double stan::model::grad\_hess\_log\_prob | ( | const M & | *model*, |
|  |  | std::vector< double > & | *params\_r*, |
|  |  | std::vector< int > & | *params\_i*, |
|  |  | std::vector< double > & | *gradient*, |
|  |  | std::vector< double > & | *hessian*, |
|  |  | std::ostream \* | *msgs* = `0` |
|  | ) |  |  |

Evaluate the log-probability, its gradient, and its Hessian at params\_r.

This default version computes the Hessian numerically by finite-differencing the gradient, at a cost of O(params\_r.size()^2).

Template Parameters
:   |  |  |
    | --- | --- |
    | propto | True if calculation is up to proportion (double-only terms dropped). |
    | jacobian\_adjust\_transform | True if the log absolute Jacobian determinant of inverse parameter transforms is added to the log probability. |
    | M | Class of model. |

Parameters
:   |  |  |
    | --- | --- |
    | model | Model. |
    | params\_r | Real-valued parameter vector. |
    | params\_i | Integer-valued parameter vector. |
    | gradient | Vector to write gradient to. |
    | hessian | Vector to write gradient to. hessian[i\*D + j] gives the element at the ith row and jth column of the Hessian (where D=params\_r.size()). |
    | msgs | Stream to which print statements in Stan programs are written, default is 0 |

Definition at line 341 of file util.hpp.

template<class M >

|  |  |  |  |
| --- | --- | --- | --- |
| void stan::model::grad\_tr\_mat\_times\_hessian | ( | const M & | *model*, |
|  |  | const Eigen::Matrix< double, Eigen::Dynamic, 1 > & | *x*, |
|  |  | const Eigen::Matrix< double, Eigen::Dynamic, Eigen::Dynamic > & | *X*, |
|  |  | Eigen::Matrix< double, Eigen::Dynamic, 1 > & | *grad\_tr\_X\_hess\_f*, |
|  |  | std::ostream \* | *msgs* = `0` |
|  | ) |  |  |

Definition at line 452 of file util.hpp.

template<class M >

|  |  |  |  |
| --- | --- | --- | --- |
| void stan::model::gradient | ( | const M & | *model*, |
|  |  | const Eigen::Matrix< double, Eigen::Dynamic, 1 > & | *x*, |
|  |  | double & | *f*, |
|  |  | Eigen::Matrix< double, Eigen::Dynamic, 1 > & | *grad\_f*, |
|  |  | std::ostream \* | *msgs* = `0` |
|  | ) |  |  |

Definition at line 405 of file util.hpp.

template<class M >

|  |  |  |  |
| --- | --- | --- | --- |
| void stan::model::gradient\_dot\_vector | ( | const M & | *model*, |
|  |  | const Eigen::Matrix< double, Eigen::Dynamic, 1 > & | *x*, |
|  |  | const Eigen::Matrix< double, Eigen::Dynamic, 1 > & | *v*, |
|  |  | double & | *f*, |
|  |  | double & | *grad\_f\_dot\_v*, |
|  |  | std::ostream \* | *msgs* = `0` |
|  | ) |  |  |

Definition at line 428 of file util.hpp.

template<class M >

|  |  |  |  |
| --- | --- | --- | --- |
| void stan::model::hessian | ( | const M & | *model*, |
|  |  | const Eigen::Matrix< double, Eigen::Dynamic, 1 > & | *x*, |
|  |  | double & | *f*, |
|  |  | Eigen::Matrix< double, Eigen::Dynamic, 1 > & | *grad\_f*, |
|  |  | Eigen::Matrix< double, Eigen::Dynamic, Eigen::Dynamic > & | *hess\_f*, |
|  |  | std::ostream \* | *msgs* = `0` |
|  | ) |  |  |

Definition at line 416 of file util.hpp.

template<class M >

|  |  |  |  |
| --- | --- | --- | --- |
| void stan::model::hessian\_times\_vector | ( | const M & | *model*, |
|  |  | const Eigen::Matrix< double, Eigen::Dynamic, 1 > & | *x*, |
|  |  | const Eigen::Matrix< double, Eigen::Dynamic, 1 > & | *v*, |
|  |  | double & | *f*, |
|  |  | Eigen::Matrix< double, Eigen::Dynamic, 1 > & | *hess\_f\_dot\_v*, |
|  |  | std::ostream \* | *msgs* = `0` |
|  | ) |  |  |

Definition at line 440 of file util.hpp.

template<bool propto, bool jacobian\_adjust\_transform, class M >

|  |  |  |  |
| --- | --- | --- | --- |
| double stan::model::log\_prob\_grad | ( | const M & | *model*, |
|  |  | std::vector< double > & | *params\_r*, |
|  |  | std::vector< int > & | *params\_i*, |
|  |  | std::vector< double > & | *gradient*, |
|  |  | std::ostream \* | *msgs* = `0` |
|  | ) |  |  |

Compute the gradient using reverse-mode automatic differentiation, writing the result into the specified gradient, using the specified perturbation.

Template Parameters
:   |  |  |
    | --- | --- |
    | propto | True if calculation is up to proportion (double-only terms dropped). |
    | jacobian\_adjust\_transform | True if the log absolute Jacobian determinant of inverse parameter transforms is added to the log probability. |
    | M | Class of model. |

Parameters
:   |  |  |  |
    | --- | --- | --- |
    | [in] | model | Model. |
    | [in] | params\_r | Real-valued parameters. |
    | [in] | params\_i | Integer-valued parameters. |
    | [out] | gradient | Vector into which gradient is written. |
    | [in,out] | msgs |  |

Definition at line 83 of file util.hpp.

template<bool propto, bool jacobian\_adjust\_transform, class M >

|  |  |  |  |
| --- | --- | --- | --- |
| double stan::model::log\_prob\_grad | ( | const M & | *model*, |
|  |  | Eigen::VectorXd & | *params\_r*, |
|  |  | Eigen::VectorXd & | *gradient*, |
|  |  | std::ostream \* | *msgs* = `0` |
|  | ) |  |  |

Compute the gradient using reverse-mode automatic differentiation, writing the result into the specified gradient, using the specified perturbation.

Template Parameters
:   |  |  |
    | --- | --- |
    | propto | True if calculation is up to proportion (double-only terms dropped). |
    | jacobian\_adjust\_transform | True if the log absolute Jacobian determinant of inverse parameter transforms is added to the log probability. |
    | M | Class of model. |

Parameters
:   |  |  |  |
    | --- | --- | --- |
    | [in] | model | Model. |
    | [in] | params\_r | Real-valued parameters. |
    | [out] | gradient | Vector into which gradient is written. |
    | [in,out] | msgs |  |

Definition at line 173 of file util.hpp.

template<bool jacobian\_adjust\_transform, class M >

|  |  |  |  |
| --- | --- | --- | --- |
| double stan::model::log\_prob\_propto | ( | const M & | *model*, |
|  |  | std::vector< double > & | *params\_r*, |
|  |  | std::vector< int > & | *params\_i*, |
|  |  | std::ostream \* | *msgs* = `0` |
|  | ) |  |  |

Helper function to calculate log probability for `double` scalars up to a proportion.

This implementation wraps the `double` values in `stan::agrad::var` and calls the model's `log_prob()` function with `propto=true` and the specified parameter for applying the Jacobian adjustment for transformed parameters.

Template Parameters
:   |  |  |
    | --- | --- |
    | propto | True if calculation is up to proportion (double-only terms dropped). |
    | jacobian\_adjust\_transform | True if the log absolute Jacobian determinant of inverse parameter transforms is added to the log probability. |
    | M | Class of model. |

Parameters
:   |  |  |  |
    | --- | --- | --- |
    | [in] | model | Model. |
    | [in] | params\_r | Real-valued parameters. |
    | [in] | params\_i | Integer-valued parameters. |
    | [in,out] | msgs |  |

Definition at line 41 of file util.hpp.

template<bool jacobian\_adjust\_transform, class M >

|  |  |  |  |
| --- | --- | --- | --- |
| double stan::model::log\_prob\_propto | ( | const M & | *model*, |
|  |  | Eigen::VectorXd & | *params\_r*, |
|  |  | std::ostream \* | *msgs* = `0` |
|  | ) |  |  |

Helper function to calculate log probability for `double` scalars up to a proportion.

This implementation wraps the `double` values in `stan::agrad::var` and calls the model's `log_prob()` function with `propto=true` and the specified parameter for applying the Jacobian adjustment for transformed parameters.

Template Parameters
:   |  |  |
    | --- | --- |
    | propto | True if calculation is up to proportion (double-only terms dropped). |
    | jacobian\_adjust\_transform | True if the log absolute Jacobian determinant of inverse parameter transforms is added to the log probability. |
    | M | Class of model. |

Parameters
:   |  |  |  |
    | --- | --- | --- |
    | [in] | model | Model. |
    | [in] | params\_r | Real-valued parameters. |
    | [in,out] | msgs |  |

Definition at line 133 of file util.hpp.

template<bool propto, bool jacobian\_adjust\_transform, class M >

|  |  |  |  |
| --- | --- | --- | --- |
| int stan::model::test\_gradients | ( | const M & | *model*, |
|  |  | std::vector< double > & | *params\_r*, |
|  |  | std::vector< int > & | *params\_i*, |
|  |  | double | *epsilon* = `1e-6`, |
|  |  | double | *error* = `1e-6`, |
|  |  | std::ostream & | *o* = `std::cout`, |
|  |  | std::ostream \* | *msgs* = `0` |
|  | ) |  |  |

Test the log\_prob\_grad() function's ability to produce accurate gradients using finite differences.

This shouldn't be necessary when using autodiff, but is useful for finding bugs in hand-written code (or agrad).

Template Parameters
:   |  |  |
    | --- | --- |
    | propto | True if calculation is up to proportion (double-only terms dropped). |
    | jacobian\_adjust\_transform | True if the log absolute Jacobian determinant of inverse parameter transforms is added to the log probability. |
    | M | Class of model. |

Parameters
:   |  |  |
    | --- | --- |
    | model | Model. |
    | params\_r | Real-valued parameter vector. |
    | params\_i | Integer-valued parameter vector. |
    | epsilon | Real-valued scalar saying how much to perturb. Defaults to 1e-6. |
    | error | Real-valued scalar saying how much error to allow. Defaults to 1e-6. |
    | o | Output stream for messages. Defaults to std::cout. |
    | msgs | Stream to which Stan programs write. Defaults to 0. |

Returns
:   number of failed gradient comparisons versus allowed error, so 0 if all gradients pass

Definition at line 268 of file util.hpp.


---

[ Stan Home Page ]

*© 2011–2014,
Stan Development Team.*
